# Dr. Adams on Periodical Blood-Letting

**Published:** 1815-08

**Authors:** 


*For the London Medical and Physical Journal.*

### Dr. Adams *on Periodical Blood-letting.*

MAY I request a few pages of your interesting Journal for a practical hint on a subject which does not seem to me sufficiently inforced by medical writers: I mean the regularity with which some persons are visited by a particular state of health at certain periods. To go through the enquiry would be to write a book of commentaries, instead of a transient essay; to enforce precepts instead of offering hints. I shall, therefore, confine myself to a few remarks on a custom now only known by tradition in most parts of the kingdom, but still within the memory of many in the remoter counties—that of annual or biennial blood-letting, or some other evacuation. That such a custom was formerly more universal than was necessary, cannot be questioned, but it is scarcely less certain, that its universality must have been the effect of some real or supposed advantage. I shall, therefore, first shew the reason why it might formerly be more necessary than at this time, which will lead to the cause of its gradual disuse; and next endeavour to prove, that even in these days there are subjects by whom this apparently antiquated practice should not be overlooked.

That the practice of blood-letting was more necessary formerly than at present appears to me accounted for, inasmuch as in proportion to the poverty and simple habits of a nation they approach nearer to what we call a state of nature. What that state really is, we have no opportunity of knowing, because we see no nation, however wild, without some mutual dependance on each other, which unites them for mutual protection or convenience. But it cannot be questioned, that civilization is favourable to the increased numbers of the human race, or that in proportion as we find them in a savage state their country is more thinly peopled.

Moralists are very apt to talk of luxury and consequent disease. It might, perhaps, be nearer the truth, if they spoke of the increased value we set on life in proportion to the number of rational enjoyments by which it is enhanced, and of our wish to have the full relish of those enjoyments which can only be accomplished by the preservation of health. These considerations render life and health greater objects of attention, and of consequence multiply physicians and remedies, and bring diseases into notice which were before either thought incurable or unattended to.

Whatever may be said of the physical strength attending a simple diet, and the privations of a savage life, it must be admitted, admitted, that security, with the temperate enjoyments of a civilized state, is favourable to health. Other animals improve by the care with which they are domesticated; still, however, they remain subject to periodical revolutions in their state of health. The horse kept constantly in the stable has less occasion, and is less provided by nature, with a change of coating according to the different seasons, but some change takes place, and that change affects the health of many of that race. The same may be said of other animals. In the spring, as this temporary sickness ceases, convalescence is followed by higher health than ordinary; and, if, at that time, an accident of any kind should induce disease, it shews itself with marks of higher action, and consequently with high inflammation. Even without any apparent cause, the predisposition to certain organic diseases existing, spontaneous inflammation may be excited by the mere increased action of all the vessels. This is so well understood, that in some of the London breweries, where the horses are always well fed, and where their value from the improved breed is considerable, it is customary to bleed the whole stud every returning spring. In the human race, this indiscriminate practice is certainly not necessary. It is only under particular circumstances that we discover any important changes at certain seasons, and, as it is always in our power to learn these, our practice should be regulated accordingly. In general, where there is a constitutional disease, or disposition to it, we shall find it exasperated or excited at certain seasons. This was so well understood, that, not much more than thirty years ago, it was the custom to bleed a considerable number of the patients of both sexes in Bedlam in the spring of the year; a custom which, I doubt not, is continued in a certain degree to this time. This practice in a hospital, the medical department of which had remained in the same family for a series of years, could only have arisen and been continued by experience of its necessity or advantage. It is evident, by a passage in the *Ars Poetica*, that, during spring, evacuations of some kind were thought necessary in Horace's time for those who were suspected of a disposition to madness. Alluding ironically to an opinion, that a degree of insanity was necessary to constitute a poet, he affects to blame himself for taking the precaution of using evacuents as the spring sets in, and thus escaping the poetical fury—

O ego lævus

Qui purgo bilem verni sub temporis horam.

Those who are subject to epilepsy, usually find their paroxysms more frequent or more severe in the spring; and and hence, it has often occurred to me, that the pretended prophecy of the augur to Julius Cæsar, who was subject to that disease, was nothing more than that he might expect a more severe return during the ides of March.

But my principal object in these remarks is, to shew the necessity of watching the return of certain seasons in those who have considerable hæmorrhage, or who have shewn symptoms of apoplexy. Though these occur most commonly in spring, yet it need not be remarked, that there is some variety, not only in the season, but the returning periods, all which should be carefully watched by the practitioner, and inculcated on the patient or his family. The necessity of bleeding in apoplexy is now so generally admitted, that I shall only take notice of one objection by the late Dr. Fothergill, in order to illustrate the subject and increase that caution which to me appears so absolutely necessary.

"Among the several causes of apoplexy, (says this, in many respects, ingenious physician) perhaps, a plentiful meal is the most common. I need only refer to the numerous instances of sudden deaths that are mentioned in the daily papers. Scarce any thing is more common than articles relating that such-a-one dropped out of his chair after eating a full meal."

The learned writer, it is well known, imputed this event to the pressure of the contents of the stomach on the descending aorta. Our improved knowledge of physiology is sufficient to satisfy us, that ample provision is made against such a consequence from such a cause. But it does not seem to me sufficiently in the constant recollection of most of us, though among the first aphorisms of our great master, that the highest health is often the forerunner of disease. Sydenham has illustrated this in a most beautiful manner, when describing the early symptoms of gout, particularly remarking that after a few days of reduced health, "the day preceding the fit, the appetite is sharp and preternatural." Thus, the hearty meal is, by this great physician, considered only as a part of, and not the cause of the paroxysm. The same frequently happens before many other acute diseases; and hence, high health occurring at a suspicious period, instead of being considered as a security, should always be suspected. The spring is well known to be favourable to high health in the animal, as well as necessary to the renovation of the vegetable world; yet, during this season, pleurisies and peripneumonies abound. It is not easy to account for the adage, "an ague in spring is fit for a king," but by the general impression that more severe diseases were appre-

 hended.

hended. Dr. Heberden remarks,* that the number of sudden deaths has doubled within the last century. Is this to be imputed, in part, to our greater freedom from other vernal diseases, and, in consequence, to the too little attention we pay to the changes some constitutions undergo during that season.

*Hatton-Garden; July 9th,* 1815. J. A.

* Commentaries of the Bills of Mortality.

